# Aflatoxin and Liver Cancer in China: The Evolving Research Landscape

**DOI:** 10.3390/toxins18020061

**Published:** 2026-01-25

**Authors:** Jian-Guo Chen, Thomas W. Kensler, Gui-Ju Sun, Jian Zhu, Jian-Hua Lu, Da Pan, Yong-Hui Zhang, John D. Groopman

**Affiliations:** 1 Department of Epidemiology, Qidong Liver Cancer Institute, Qidong People’s Hospital, Affiliated Qidong Hospital of Nantong University, Qidong 226200, China; jsqdzj8888@sina.com (J.Z.); jianhualu3@163.com (J.-H.L.); qdzyh680@aliyun.com (Y.-H.Z.); 2 Department of Environmental Health and Engineering, Johns Hopkins Bloomberg School of Public Health, Baltimore, MD 21205, USA; 3 Key Laboratory of Environmental Medicine and Engineering of Ministry of Education, Department of Nutrition and Food Hygiene, School of Public Health, Southeast University, Nanjing 210009, China; gjsun@seu.edu.cn (G.-J.S.); pan_da@seu.edu.cn (D.P.)

**Keywords:** aflatoxin, hepatocellular carcinoma, molecular epidemiology, TP53 R249S, chemoprevention, exposure assessment, food safety standards

## Abstract

Aflatoxins, particularly aflatoxin B_1_ (AFB_1_), are among the most potent naturally occurring carcinogens and remain a major food-borne hazard in parts of Asia and Africa. China has generated a uniquely cohesive body of evidence connecting aflatoxin contamination to hepatocellular carcinoma (HCC), especially in settings where chronic hepatitis B virus (HBV) infection is highly prevalent and acts synergistically with aflatoxin exposure. Over five decades, field investigations and laboratory innovations—exemplified by long-term work in Qidong—have assembled a multi-layered causal chain spanning the following: (i) contamination monitoring in staple foods; (ii) quantification of internal dose and biologically effective dose using validated biomarkers (e.g., urinary AFB_1_–N^7^–guanine, AFM_1_, and serum AFB_1_–lysine albumin adducts); (iii) a characteristic molecular fingerprint in tumors and circulation (TP53 R249S); (iv) reversibility demonstrated through randomized intervention trials and policy-driven natural experiments. Chemoprevention and dietary interception studies (e.g., oltipraz, chlorophyllin, and broccoli sprout beverages) showed that enhancing detoxication pathways can lower biomarker burdens in exposed populations. At the population level, a sustained dietary transition from maize to rice, together with strengthened food governance, was accompanied by marked decreases in biomarker distributions and subsequent declines in HCC mortality in endemic regions. Nevertheless, regional heterogeneity, multi-mycotoxin co-exposure, and climate variability are expected to increase exposure volatility and complicate surveillance. Here, we translate and synthesize the Chinese evidence base, highlight biomarker-enabled monitoring and policy evaluation, and propose an integrated “5+1” prevention framework spanning source control, process detoxification, tiered governance, short-course interception, precision follow-up of high-risk individuals, and climate-sensitive early warning along the climate–agriculture–storage–processing–population (CAT–CSPP) chain.

## 1. Origins of the Research: From the Laboratory to the Field

Since the 1970s, recurrent mold spoilage in grains and edible oils in the Yangtze River Delta and humid subtropical southern China—together with sporadic livestock and poultry toxicosis—has raised concern about a link between aflatoxin (AFB_1_) exposure and localized clusters of high HCC incidence [1,2,3,4,5]. Field investigations in Qidong and coastal townships of Nantong subsequently established an evidentiary thread—“mold-contaminated staples → experimental carcinogenesis → elevated population incidence”—and framed storage conditions, feed–related toxicosis, and human tumor occurrence within a unified “exposure → biological effect → health outcome” pathway [3,4,5,6,7,8].

Methodological breakthroughs then advanced the evidence chain into quantifiable exposure. In 1985, a study on highly exposed populations in Guangxi reported the first simultaneous urinary detection of the major aflatoxin–DNA adduct (AFB_1_–N^7^–Gua) and the metabolites (AFM_1_, and AFP_1_), demonstrating that very-low-dose human exposure can be captured by biomarkers [9]. Subsequent work in molecular dosimetry studies showed a dose–response relationship between dietary AFB_1_ intake and urinary excretion of AFB_1_–DNA adducts, enabling translation from laboratory measures to population risk assessment [10,11,12]. Building on these advances, a prospective nested case–control study of 18,244 men in Shanghai demonstrated that detectable baseline urinary AFB_1_–N^7^–Gua predicted increased HCC risk and interacted multiplicatively with HBsAg positivity; extended follow-up analyses yielded consistent results [13]. Collectively, these studies marked the transition from “detectable exposure” to “predictive risk” in population-based research in China.

Molecular pathological evidence emerged in parallel. In HCC from Qidong, where aflatoxin exposure is historically dominant, the high frequency of the TP53 codon-249 AGG → AGT (R249S) hotspot mutation provided a fingerprint-like clue linking AFB_1_ to tumorigenesis [14]. This signal was later supported at broader scales: R249S was detected in tumor tissues and circulating DNA in high-exposure populations and showed geographic clustering that broadly paralleled local AFB_1_ contamination in grains/oils and HBV prevalence [15,16,17]. Together, toxicologic and molecular epidemiologic evidence strengthened the shift from general “statistical association” to “mechanistic specificity.”

**Figure 1:**
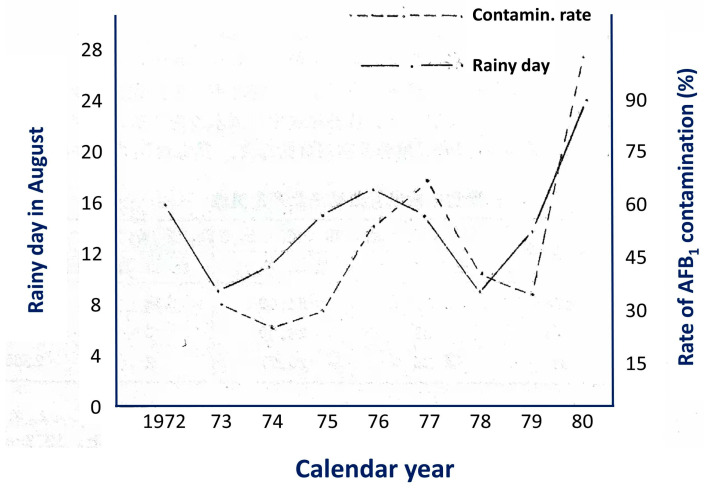
August rainy days and aflatoxin B_1_ (AFB_1_) contamination rate in maize, Qidong (1972–1980). Two annual time series are displayed with dual y-axes: August rainy days (left y-axis) and the percentage of AFB_1_-contaminated corn samples (right y-axis). Data are aggregated by calendar year. Adapted from reference [3].

From the late 1990s into the early 21st century, laboratory evidence was translated into field-based intervention. Community trials in Qidong showed that oltipraz and chlorophyllin/chlorophyll-based intervention could reduce key biomarkers, including urinary AFB_1_–N^7^–Gua and serum AFB_1_–albumin adducts, with good feasibility and adherence [18,19]. At the population level, Qidong’s dietary structure prior to the mid-1980s relied heavily on maize as the staple, with maize spoilage shaped by local climatic conditions (Figure 1). After the mid-1980s, as the staple diet shifted naturally from mold-prone maize to rice, monitoring documented an order-of-magnitude decline in internal exposure. Correspondingly, age-standardized HCC incidence/mortality in the region showed a marked and sustained decline after the 1990s, constituting a rare population-scale causal chain consistent with “declining exposure → declining disease burden” [20,21]. This “natural experiment” strengthened etiologic attribution of AFB_1_ and provided a public-health template for upstream risk control across harvesting, storage, transport, and processing of susceptible commodities.

Overall, China’s experience illustrates how biomarker-enabled surveillance and targeted interventions can support measurable risk reduction and policy evaluation in high-exposure settings, while informing adaptive strategies for climate- and supply chain-driven exposure volatility.

## 2. Pillars of Evidence: From Biomarkers to Etiology

Chinese research has anchored the AFB_1_ → HCC causal chain on three mutually reinforcing pillars: (i) quantifiable human exposure; (ii) biologically plausible and epidemiologically demonstrable interaction with HBV infection; (iii) a specific molecular signature in tumors and circulation. Importantly, intervention trials have provided ‘reversibility’ evidence, showing that lowering internal dose can lower markers of biologically effective dose.

### 2.1. Prospective Population Evidence: Dose–Response and Synergy with HBV

With methodological maturation, biomarker measurements were brought into field-based studies. In populations from southern China, multiple urinary biomarkers—AFB_1_–N^7^–Gua, AFM_1_, and AFP_1_—could be detected simultaneously, and their levels were positively correlated with short-term dietary AFB_1_ intake, supporting their utility as indicators of recent exposure [9,10]. A prospective nested case–control study among 18,244 men in Shanghai further showed that individuals positive for baseline urinary AFB_1_–DNA adducts had a significantly higher subsequent HCC risk. When HBsAg positivity co-occurred with high-level AFB_1_ exposure, risk increased in a multiplicative (and potentially supra-multiplicative) manner, consistent with strong synergy rather than simple additivity [13].

In longitudinal follow-up in Daxin (Daxing), Qidong, serum AFB_1_–albumin adduct levels varied with seasonality and fluctuations in grain sources, indicating a population exposure pattern that is chronic, low-dose, yet dynamically variable. Notably, HBsAg-positive individuals with persistent long-term adduct positivity were more likely to develop subsequent tumor-related abnormalities, reinforcing the field-based concept that “measurable exposure” can translate into “predictable risk” [22]. Collectively, these findings form a population evidence base supporting the transition from quantifiable internal dose to risk prediction. Accurate quantification of dietary AFB_1_ intake at the individual level is inherently challenging in population studies because contamination in staple foods is highly heterogeneous across batches, storage conditions, and seasons, and because daily consumption is often estimated from questionnaires with unavoidable measurement error [23]. Therefore, direct intake estimates alone are unlikely to capture true internal dose, especially in large cohorts. In this context, biomarker-based exposure assessment (e.g., urinary AFB_1_–N^7^–Gua/AFM_1_/AFP_1_ and serum AFB_1_–albumin adducts) provides an accepted and relevant alternative because it integrates exposure from multiple food sources over defined time windows and more directly reflects internal and biologically effective doses [24,25]. Accordingly, throughout this review, we emphasize biomarker evidence as the most actionable approach for exposure–risk inference and for evaluating interventions when precise daily intake cannot be reliably measured.

Although this review focuses on the synergistic effect of chronic hepatitis B virus (HBV) infection and AFB_1_ exposure—because this interaction is supported by the most complete China-based evidence chain (biomarkers, molecular fingerprints, and intervention/natural-experiment data)—hepatitis C virus (HCV) infection is also a well-recognized etiologic factor for HCC and contributes to HCC risk in China. In practice, national and large-scale prevention efforts in China have historically placed greater emphasis on HBV-related control and monitoring, whereas widely accessible nationwide data resources on HCV prevalence and long-term follow-up, as well as studies explicitly evaluating potential AFB_1_–HCV joint effects, are comparatively fewer than those for HBV in the public literature. Accordingly, future work should further evaluate whether and how aflatoxin-related risks interact with HCV in specific settings.

### 2.2. Molecular Fingerprint Evidence: TP53 R249S as an Etiologic ‘Nail’

In terms of etiologic specificity, the TP53 codon-249 AGG → AGT (R249S) mutation has been established as a molecular fingerprint of HCC in regions with high AFB_1_ exposure [14,15,17,26]. The mutation is detected at markedly higher frequency in tumor tissues from high-exposure areas of southern China, and its prevalence shows geographic concordance with regional exposure gradients, strengthening the spatial linkage between aflatoxin contamination and characteristic mutational profiles.

Crucially, R249S is not confined to tumor tissue. It can be prospectively captured in plasma cell-free DNA among individuals without a clinical diagnosis of HCC at baseline, and carriers of circulating R249S show a significantly increased subsequent risk of developing HCC. This provides time-sequence evidence connecting “early exposure signals,” “early molecular events,” and “tumor occurrence” [27,28]. Independent studies in Guangxi and Qidong have reproduced this chain of evidence, reinforcing external consistency and reproducibility across populations and exposure settings [29,30].

### 2.3. Intervention and Reversibility Evidence: From Measurable Exposure to Modifiable Risk

Whether etiologic evidence can be translated into reversible risk is central to its public-health value. Human intervention trials conducted in Qidong provided proof that aflatoxin-related internal dose can be reduced through mechanism-based strategies. In community studies, oral oltipraz was shown to inhibit phase I activation of AFB_1_ while inducing phase II detoxification, resulting in significant reductions in urinary AFB_1_–N^7^–Gua and serum AFB_1_–albumin adducts [18]. In a subsequent double-blind randomized trial, chlorophyllin (100 mg, three times daily for 4 months) significantly decreased the urinary excretion of AFB_1_–DNA adducts, supporting a chemopreventive effect through interception and reduced DNA binding of the carcinogen [19].

**Figure 2:**
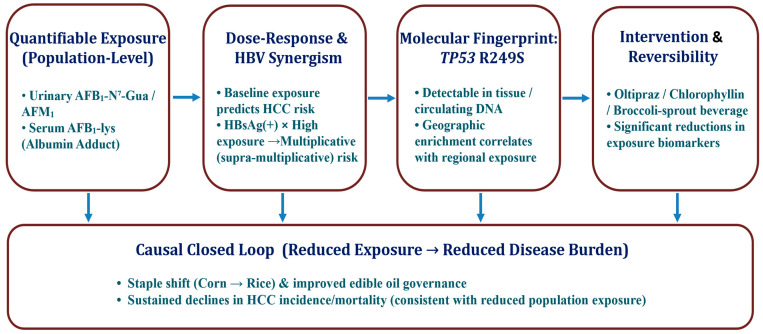
From biomarkers to causation: evidence pillars for aflatoxin B_1_ → HCC (China).

Later, a food-based approach using broccoli sprout beverages as a dietary intervention also enhanced detoxification and excretion of electrophilic intermediates in Chinese populations, offering a more scalable “food–nutrition” pathway for exposure mitigation [31,32]. By using biomarker modulation as a surrogate endpoint in place of long-latency cancer outcomes, these trials delivered short-term, reproducible effects in high-exposure groups that aligned directionally with the cohort and molecular pathology evidence, thereby forming a closed loop of “measurable–controllable–evaluable” prevention. The main pillars of this evidence chain are summarized in Figure 2**.**

Schematic summarizing three evidence pillars linking aflatoxin B_1_ (AFB_1_) exposure to hepatocellular carcinoma (HCC) in China: (1) prospective biomarker-based epidemiology with dose–response and strong synergy with HBsAg (HBV); (2) the TP53 R249S molecular fingerprint establishing exposure specificity and temporality (including detection in circulating DNA prior to diagnosis); (3) intervention and policy evidence demonstrating reversibility of biomarker levels and reductions in population burden (e.g., oltipraz, chlorophyllin, broccoli sprout beverage; corn → rice dietary shift). A bottom pipeline connects measurable exposure (AFB_1_–lys, AFB_1_–N^7^–Gua, AFM_1_) to causal inference and actionable prevention. (Key refs: [12,13,14,18,19,20,21])

## 3. Biomarker Applications: From ‘Measurable’ to ‘Usable’

The distinctive contribution of the Chinese program lies in converting laboratory biomarkers into operational tools that can be deployed in field settings, compared across years, and used to evaluate interventions and policy. This work addressed both ‘can we measure accurately?’ (sensitivity, specificity, matrix effects) and ‘can we use scientifically?’ (population monitoring, risk stratification, follow-up, and governance feedback loops).

### 3.1. Translating TP53 R249S into Applied Molecular Etiology

The TP53 codon-249 AGG → AGT substitution (R249S) has become a molecular “fingerprint” for aflatoxin-related hepatocellular carcinoma (HCC). It was first reported in tumor tissues from Chinese high-incidence areas (Qidong) by Hsu et al. [14], and it was subsequently shown by Aguilar et al. [15]—through geographic comparisons—to be significantly enriched in regions with high AFB_1_ exposure, supporting a robust exposure–mutation correspondence at the population level [26,33,34]. The key step that transformed R249S from an intriguing tumor signature into an actionable etiologic indicator was the field-oriented evolution of assays: early tissue-based methods (e.g., PCR with restriction enzyme-based approaches and hybridization assays) were progressively complemented by high-sensitivity platforms capable of detecting very low-abundance mutant fragments in plasma/circulating cell-free DNA. This methodological shift enabled prospective capture of R249S in individuals who had not yet developed clinically diagnosed HCC but who were in the etiologically relevant background of HBV positivity plus aflatoxin exposure, thereby linking molecular attribution to real-world surveillance and early warning.

Importantly, multiple cohorts and studies from Qidong, Guangxi, and areas around Shanghai reproduced this signal at both the tumor tissue and circulating DNA levels, and further supported a temporally coherent relationship in which R249S positivity precedes and predicts subsequent HCC occurrence [12,27,30]. Nevertheless, the detection rate of R249S is influenced by the spatiotemporal intensity of aflatoxin exposure and by HBV replication dynamics, and it is not a necessary condition for every aflatoxin-related HCC. Its practical role is therefore best framed as an etiologic indicator and high-risk signal, rather than a universal diagnostic hallmark. In population prevention, the fundamental strategy remains reducing overall AFB_1_ exposure, with R249S serving as a complementary tool for etiologic attribution and risk stratification when interpreted alongside exposure biomarkers and HBV markers [14,15,26,30].

### 3.2. Protein Adducts and Isotope-Dilution LC–MS/MS: Comparability and Time-Series Monitoring

Urinary biomarkers such as AFM_1_ and AFB_1_–N^7^–Gua capture short-window exposure and are readily influenced by day-to-day dietary variation. In contrast, the serum albumin AFB_1_–lysine adduct (AFB_1_–lys) integrates exposure over approximately 2–3 months, making it more suitable for cross-seasonal and inter-annual monitoring. Around the early 2000s, China established laboratory workflows aligned with international practice in multiple provinces, incorporating immunoaffinity enrichment, enzymatic digestion to release AFB_1_–lys, and isotope-dilution LC–MS/MS quantification [20,35,36]. These platforms achieved limits of detection on the order of 0.1–0.3 pg/mg albumin, that would translate to daily exposures of 70 ng aflatoxin, thus fitting contemporary settings in which population exposure has markedly declined [37].

Pooled re-analyses across countries further indicate that normalizing AFB_1_–lys to serum albumin concentration can substantially improve interpretability and comparability across studies and regions [38]. This point is particularly important for China: only under a unified, traceable methodological framework can the high exposures typical of maize/peanut-based diets in the 1980s be compared on the same scale with the lower exposures observed after 2000 as rice became the dominant staple. Such comparability is essential for rigorously evaluating the real-world impact of socioeconomic and dietary transitions, as well as improvements in regulation and grain storage practices, on reducing aflatoxin exposure [9,13,20,21].

### 3.3. Scenario-Based Deployment: Surveillance Networks and Intervention Evaluation

(1) Surveillance

At the CDC or hospital-laboratory level, domestically produced monoclonal antibodies combined with disposable affinity columns and HPLC/fluorescence can be used for initial screening of edible oils/maize and serum samples. Samples with suspicious results or high values can then be forwarded to provincial platforms for isotope-dilution LC–MS/MS confirmation, forming a tiered network of “field sampling → regional screening → central confirmation → result feedback” [39].

(2) Follow-up and risk stratification

In regions with high HBV endemicity and non-negligible AFB_1_ risk, a stepwise pathway can be implemented—HBsAg screening → annual AFB_1_–lys testing → additional R249S testing for persistently high AFB_1_–lys—to embed biomarker-defined exposure into high-risk population management.

(3) Intervention evaluation

Chemopreventive and nutritional interventions exemplified by Qidong were operationalized through a model of village/township sampling, centralized testing, and phased feedback. Randomized controlled trials of oltipraz and chlorophyllin demonstrated the modifiability of internal AFB_1_ metabolism via phase I inhibition/phase II induction, and broccoli sprout beverages (rich in glucoraphanin/sulforaphane) likewise significantly enhanced electrophile detoxification and elimination in human populations [18,19,29].

(4) Policy evaluation

Following the structural dietary transition from maize to rice, multi-year monitoring showed an overall downward shift in the population distribution of AFB_1_–lys, with a marked compression of the high-exposure tail. This biomarker trend paralleled the long-term decline in age-standardized HCC incidence/mortality in the same settings, supporting a population-level “policy–exposure–outcome” linkage [20,21].

### 3.4. Bidirectional Linkage Between Methods and Governance

Operational “usability” depends not only on analytical sensitivity, but also on standardization that interfaces with governance and risk management. First, reporting should include key performance characteristics—limit of detection (LOD) and limit of quantification (LOQ), recovery, and intra-/inter-day relative standard deviations (RSDs)—to support method transparency and comparability [40]. For cross-region comparisons, AFB_1_–lys should be reported in parallel as both the absolute value and the albumin-normalized value, enabling independent verification and more consistent interpretation across studies and laboratories [38]. Second, surveillance findings must be translated rapidly through risk communication, risk-based sampling and inspection tiers, and storage/processing improvements within grain and edible-oil supply chains—especially for long-tail producers (e.g., small workshops and small-scale oil presses)—to create a closed loop from measurement to detoxification and exposure reduction. Under this framework, the biomarker system has evolved from “proving causality” to a practical toolkit for guiding interventions and evaluating policies.

Conceptually, R249S provides etiologic specificity, AFB_1_–lys supplies a comparable time-integrated internal dose, and randomized trials together with population “natural experiments” allow declines in biomarkers to be triangulated with declines in disease burden. China’s distinctive contribution has been to connect these components into a system that can operate in real populations: it captures temporal fluctuations at surveillance sentinels while also delivering measurable endpoints for interventions and policies—thereby achieving both methodological and public-health implementation from “measurable” to “usable.”

## 4. The Qidong Experience: From Chemoprevention to Policy Intervention

Qidong’s distinctive contribution extends beyond completing three landmark human chemoprevention/dietary-interception trials. More importantly, it offers a replicable public-health paradigm characterized by a “30–50-year longitudinal observation → sustained declines in biomarker-defined exposure → concordant declines in liver cancer incidence/mortality → policy replicability” evidence chain [6,20,21]. In other words, the AFB_1_ carcinogenic pathway first established in the laboratory has been systematically validated over a long time horizon in a real, county-level population, and its implications have further been translated into actionable public-health governance with practical external relevance.

### 4.1. The ‘Validation Phase’: Chemoprevention and Dietary Interception

Qidong’s decision to pursue population-based chemoprevention was motivated by the historical co-occurrence of a high prevalence of HBsAg positivity and sustained AFB_1_ exposure driven by long-term consumption of maize and peanuts (including peanut oil). Randomized controlled studies showed that oltipraz, by suppressing phase I metabolic activation of AFB_1_ while inducing phase II detoxification, significantly reduced key biomarkers including urinary AFB_1_–N^7^–Gua and serum AFB_1_–albumin adducts. Although intermittent high-dose and continuous low-dose regimens differed somewhat in their biomarker response profiles, the direction of effect was consistent across dosing strategies [18].

A subsequent double-blind randomized trial of chlorophyllin (100 mg per dose, three times daily for 4 months) further demonstrated that, in highly exposed populations, the median adduct burden could be reduced by approximately one-half, with good safety and adherence [19]. The broccoli sprout beverage intervention—characterized by high levels of glucoraphanin/sulforaphane—translated phase II induction into a low-cost, acceptable food-based approach; studies in Chinese populations similarly observed enhanced electrophile clearance and increased urinary excretion of relevant detoxication metabolites [32]. Together, these three classes of interventions completed a critical transition from “measurable exposure” to “reduced internal dose,” and provided a technical foundation for subsequent short-cycle, repeatable evaluations that use biomarkers as practical endpoints in community prevention programs.

### 4.2. Natural Experiments as Population-Level Evidence

In parallel with randomized trials, Qidong underwent a systemic transition in dietary structure and grain/oil supply beginning in the late 1980s. Staple foods shifted from mold-prone maize to rice, while commercialization of finished grain and edible oils as well as improvements in storage conditions proceeded in tandem. Based on multi-year population monitoring, the distribution of AFB_1_–albumin adducts shifted downward overall, and the high-exposure tail was markedly “compressed,” a pattern that aligned with the sustained decline in age-standardized liver cancer mortality observed over subsequent spatial–temporal scales in the same setting [20].

A longer, ~50-year perspective further indicates that a systematic reduction in AFB_1_ exposure constituted an indispensable component of Qidong’s declining liver cancer burden. At the same time, potential co-contributors—such as improved drinking-water safety, better grain/oil quality, expanded HBV vaccination, and strengthened secondary prevention—may have added incremental benefits on top of the exposure reduction [21]. Taken together, this pathway forms a “environment/policy → exposure → outcome” triangulation, providing rare county-level population evidence for an environmentally removable cause of cancer and the feasibility of primary prevention through food-system change [20,21].

### 4.3. Key Features for Replication

The Qidong experience can be distilled into a practical four-step pathway for translation and scale-up. (1) Establish the baseline: conduct synchronized measurements of contamination in grains/foods and population biomarkers—preferably AFB_1_–lys (or urinary AFB_1_–N^7^–Gua)—to identify the major risk points by crop type, seasonality, and supply channels. (2) Implement substitution and mold control: promote mold-resistant staple substitutions and standardized storage/drying/temperature-controlled logistics, while restricting highly contaminated oil from reaching households. (3) Embed biomarker follow-up: repeat core biomarker testing at the population level every 2–3 years to quantify overall downward shifts in exposure and the convergence (“compression”) of the high-exposure tail. (4) Precision secondary prevention: among HBsAg-positive individuals with persistently high AFB_1_–lys, strengthen ultrasound/AFP surveillance and referral management to enhance early detection and timely care.

Coupled with randomized trials, this operational workflow can demonstrate short-term reductions in internal dose while enabling mid- to long-term evaluation of disease outcomes, thereby translating the health value of “mold prevention and detoxification” into an evidence base compatible with fiscal decision-making and regulatory cost–benefit considerations [18,19,20,21,29].

### 4.4. Mutual Reinforcement of Etiology and Governance

From the standpoint of causal inference, the Qidong experience consolidated the etiologic role of AFB_1_ in local HCC through a macro-level trajectory in which declining exposure coincided with declining disease burden. At the same time, by demonstrating that exposure could be substantially reduced through policy-relevant measures, Qidong reframed AFB_1_ from an ostensibly “immutable environmental hazard” into a public-health governance target that is intervenable, quantifiable, and evaluable.

This dual reinforcement helps explain why many international reviews and retrospectives cite Qidong as a prototypical setting that combines a “natural experiment” with a chemoprevention demonstration: few chemical carcinogens have generated a coherent, decades-long, county-scale time series that can be triangulated within a closed evidentiary loop spanning mechanistic, population, and policy layers [20,21,32].

### 4.5. Policy Implications Beyond a Single Region

Building on the Qidong experience and today’s regulatory and monitoring capacity, four complementary strategies can be proposed for aflatoxin control on a larger scale. (1) Upstream mold prevention: prioritize cultivar improvement, low-water activity storage, graded drying, and temperature-controlled logistics, with preferential coverage of high-risk belts in humid subtropical cropping and storage regions. (2) Midstream detoxification and interception: introduce quantitative thresholds and closed-loop recordkeeping into oil refining, grain grading/marketing, and interception of problematic batches, leveraging routine in-house testing complemented by third-party confirmation. (3) Population exposure reduction: promote dietary patterns rich in cruciferous vegetables and dark leafy greens, and consider short-course intensified interventions during seasonal high-risk periods or for specific groups (e.g., preconception/pregnancy, and HBV carriers) [32]. (4) Harmonized monitoring and evaluation: adopt AFB_1_–lys and/or urinary AFB_1_–N^7^–Gua as core indicators, standardize monitoring reports, and encourage cross-regional measurements using comparable methods and standards to build time series and spatial maps of population exposure—thereby supporting risk-based governance and resource allocation [20,21].

Overall, Qidong completed a three-in-one translation from laboratory evidence to county-level governance across methods, population epidemiology, and policy: randomized controlled trials demonstrated that internal dose can be reduced, natural experiments and long-term monitoring supported that disease burden can decline, and institutionalized workflows plus standardized measurement help ensure that gains can be replicated and evaluated. This paradigm offers a clear technology–management–evaluation pathway for other aflatoxin-prone grain/oil regions in China and provides an operational foundation for sustaining low population AFB_1_ exposure and reducing the attributable liver cancer burden under future scenarios in which climate-related mold risks may intensify [20,21,32].

## 5. Strengthening Domestic Surveillance: Exposure Assessment and Regional Features

Over the past decade, provinces and municipalities across China have continued to expand domestic surveillance programs centered on key pathways—peanut/maize → edible oils → region-specific specialty foods → dairy products. Methodologically, monitoring has advanced from routine HPLC toward immunoaffinity cleanup coupled with LC–MS/MS, improving sensitivity and specificity for low-level contamination and enabling more reliable inter-laboratory comparison. On the assessment side, these efforts increasingly incorporate probabilistic exposure modeling, P95 (high-percentile) population analyses, and margin-of-exposure (MOE)-based risk characterization [41,42,43]. Together, these approaches make it measurable and comparable to identify which product categories, which points along the supply chain, which seasons, and which population groups experience the highest exposure, thereby providing a quantitative foundation for zoned governance and policy evaluation.

### 5.1. Grain- and Oil-Dominated Exposure Pathways: From Small Oil Presses to Multi-Matrix Integration

Across provincial surveillance datasets, peanuts and peanut products, maize and maize products, and edible vegetable oils produced by small-scale pressing remain the dominant dietary sources of AFB_1_ exposure in China. In particular, traditionally pressed bulk peanut oil often exhibits both a higher contamination positivity rate and a markedly right-skewed “high-tail” intake distribution, driven by inadequate raw-material sorting, suboptimal drying and storage, and relatively rudimentary pressing practices. This pattern can make overall exposure for the general population appear “acceptable,” while high-consumption subgroups—especially those preferentially consuming locally produced bulk oils—may face substantially elevated P95 or P97.5 exposure levels, warranting targeted interventions [23,38,44,45].

In contrast to earlier monitoring focused on single commodities (e.g., oils alone), recent provincial and municipal programs increasingly adopt multi-matrix designs. These commonly cover bulk peanuts/peanut products and maize flour/maize products simultaneously, and they expand the exposure inventory to include fermented teas, dried chili peppers, and selected condiments, reflecting a “low-dose, high-frequency, long-term accumulation” pattern. Nonparametric and/or Monte Carlo methods are then used to derive MOE or quantitative risk estimates, helping identify priority intersections of food category × population subgroup × season for risk-based control [42].

**Figure 3:**
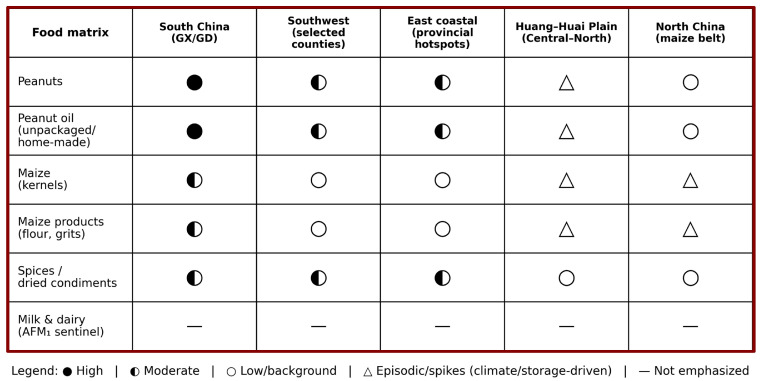
Qualitative occurrence patterns of aflatoxins across major food matrices and representative regions in China.

For milk and dairy products, surveillance often uses AFM_1_ as an entry point and applies a tiered strategy—rapid screening by ELISA or immunochromatographic tests, followed by LC–MS/MS confirmation—especially during peak milk-production periods or under cross-province distribution, to manage fluctuations attributable to silage mismanagement or extreme-weather years [24,46]. Overall, the value of a “multi-matrix synchronous assessment” framework is that it more faithfully reconstructs real-world exposure as multi-source, multi-frequency, low-dose intake, thereby providing an actionable evidence base for “combined governance” integrating upstream mold control, process-level detoxification, as well as endpoint inspection and enforcement [24,38,42,44,45]. To provide an at-a-glance synthesis of this multi-matrix perspective, Figure 3 summarizes the qualitative occurrence patterns of aflatoxins across major food matrices and representative regions in China, thereby guiding surveillance prioritization and risk communication.

Sampling and representativeness are often the dominant sources of uncertainty in aflatoxin surveillance because contamination is highly heterogeneous (“hotspots”) within lots. Thus, analytically correct determination does not guarantee accurate exposure characterization unless sampling is appropriately designed. In practice, key principles include the following: (i) clear definition of lot/sublot and incremental sampling across the lot; (ii) sufficiently large aggregate sample mass; (iii) comminution and homogenization followed by controlled subsampling to minimize particle-size effects; (iv) quality management procedures for sampling and sample preparation. Internationally, Codex provides commodity-oriented sampling approaches for aflatoxins (e.g., enforcement/control sampling plans for peanuts in CXS 193-1995, Annex 1), and ISO 24333 specifies sampling requirements for cereals and cereal products, explicitly applicable to heterogeneously distributed contaminants—both serving as practical benchmarks for surveillance and inspection program design [47,48].

### 5.2. Regional Heterogeneity and Susceptibility: “High in the South, Rising in the North” and Compound Risk in Southwest/Coastal Belts

China’s spatial pattern increasingly reflects a “high in the south, rising in the north” profile. While the southern and southwestern belt (including classic hotspot in Guangxi/Guangdong and parts of Southwest China), as well as some eastern coastal provinces, remain a major high-risk arena, multi-province surveillance indicates that during hot–dry years and other extreme weather conditions, mold spoilage and aflatoxin risk in maize- and peanut-related supply chains can rise substantially in parts of the Huang–Huai Plain and North China, underscoring that “the north is not inherently a safe zone” [49]. To make this heterogeneity explicit, we now provide a comparative national table (Table 1) summarizing, across major regions/provinces, the dominant exposure matrices, qualitative contamination patterns (high/moderate/low/episodic), availability of human biomarker evidence, and the HCC burden/co-factor context together with the corresponding evidence types (surveillance vs. cohort biomarker studies vs. intervention/policy evidence).

In areas characterized by high temperature and humidity, a high density of small-scale production/processing (e.g., small workshops and informal oil pressing), and higher HBV carriage, an “exposure–susceptibility” overlap belt may emerge (e.g., parts of Guangxi/Guangdong and selected county-level settings in Southwest China). These settings warrant more frequent product testing and more intensive population follow-up to manage seasonal and year-to-year variability. Notably, although chemoprevention and short-course dietary interception trials with biomarker endpoints have been most comprehensively conducted in Qidong and a limited number of other high-exposure settings, many provinces beyond these sentinel sites have emphasized food chain governance, risk-based inspection, and targeted surveillance rather than population-wide intervention trials. Therefore, when interpreting regional differences, it is important to distinguish between (i) regions with long-term “exposure → biomarker → intervention/policy” evidence chains and (ii) regions where the evidence is primarily surveillance-based (Table 1).

Regarding population heterogeneity, priority groups include HBsAg-positive individuals, patients with chronic liver disease, preschool children (with higher vulnerability to AFM_1_), and rural older adults who preferentially consume locally produced oils. For a given level of AFB_1_ exposure, these groups may experience greater health risk, so regional exposure assessment and risk communication should be stratified by population subgroup. In practice, P95/P97.5 percentiles should be routinely reported and considered as reference points for regulatory action and targeted intervention, rather than relying only on average exposure estimates [20,21,24,38,44,45,50,51].

### 5.3. Co-Exposure and Synergistic Toxicity: Supporting Regulation with a “Mixture/Combined-Indicator” Framework

Real-world dietary exposure is rarely limited to a single toxin such as AFB_1_. In many regions, local and university laboratories frequently detect co-occurring mixtures in the same samples—most commonly AFB_1_ + fumonisin B_1_ (FB_1_) + zearalenone (ZEN)—reflecting the ecological reality that multiple fungal species can coexist along storage, transport, and warehousing chains [52]. Experimental evidence from cell and animal models further suggests that AFB_1_ may display additive or synergistic toxicity when combined with FB_1_ and/or ZEN, and that such mixtures can perturb hepatocellular transcriptomic programs, including changes in alternative splicing [25,54].

These observations imply that risk characterization based solely on single-compound limits may underestimate the true population burden in settings where co-contamination is common. Accordingly, several Chinese assessments have adopted nonparametric probabilistic approaches and Monte Carlo simulations to integrate low-dose, high-frequency intake across multiple food categories, providing “worst-case” scenarios alongside uncertainty bounds or credible intervals. This practice aligns with the broader co-exposure assessment logic used by agencies such as EFSA and supports the idea that standard revisions and inspection schemes should treat commonly co-occurring mycotoxin combinations as pragmatic regulatory units, improving the consistency of testing, decision thresholds, and risk communication with real-world exposure patterns [38,58].

### 5.4. Monitoring Systems and Methodological Details: From “Measurable” to “Accurate, Frequent, and Transparent”

Recent domestic progress is most evident in three areas.

(1) Standardized sample preparation and analytical platforms. Immunoaffinity columns (IAC), dispersive solid-phase extraction (d-SPE), and QuEChERS have been developed in parallel and, when coupled with isotope-dilution LC–MS/MS, have improved recovery, lowered detection limits, and increased batch throughput. Reporting practices increasingly include LOD/LOQ, recovery, and intra-/inter-day RSD, and for cross-regional comparisons of AFB_1_–lys, it is advisable to report both the non-normalized value and the albumin-normalized value to enhance comparability and interpretability [38].
(2) A two-tier “screening–confirmation” network. During key windows—such as peak milk-production periods, seasonal transitions, and post-typhoon or moisture-rebound episodes—frontline screening by ELISA or immunochromatographic assays can expand coverage, while positive or suspicious samples are routed to LC–MS/MS for confirmation. For oils and grains, municipal laboratories can undertake initial screening and repeat sampling, whereas provincial platforms provide harmonized confirmation and proficiency assessment, forming a closed loop of “field sampling → regional screening → central confirmation → feedback” [55].
(3) Risk-tiered sampling targets and frequencies. A rolling priority list can be built around “priority matrices × priority regions × priority actors” (e.g., locally pressed oils, bulk peanuts, dried chili/chili powder, and fermented teas), with increased sampling frequency during high-risk seasons and extreme-weather years. In parallel, making a given supply chain traceable as a time series allows assessment of how storage, drying, long-distance transport, and re-processing contribute to contamination dynamics. For example, “high-temperature drying + long-haul transport” can be explicitly incorporated into risk identification and inspection plans for dried chili/chili powder [42,44,45,46,51].

### 5.5. Linking Exposure Surveillance to Health Outcomes and Policy Evaluation

Connecting domestic surveillance with population evidence and policy interventions is essential for an integrated “monitoring → governance → impact evaluation” framework. Methodologically, using AFB_1_–lys as a time-integrated population dose metric together with urinary AFB_1_–N^7^–Gua as a short-window exposure indicator allows dual use as follows: (i) risk-stratified management in county-level follow-up (e.g., intensified ultrasound/AFP surveillance for individuals with HBsAg positivity plus persistently high AFB_1_–lys); (ii) before–after evaluation of policy actions such as the “maize → rice” dietary transition and improvements in storage systems. If the population mean shifts downward and the right-tail distribution converges, and these biomarker trends align with long-term declines in HCC incidence/mortality, the resulting evidence can provide strong quantitative support for resource allocation and enforcement of food safety standards [20,21,38].

From a national perspective, surveillance-informed zoned governance (e.g., humid southern belts, the Huang–Huai grain-producing belt, and hot–dry northern belts) combined with a priority matrix of susceptible groups (high-HBV-prevalence communities, preschool children, rural older adults, and consumers of locally pressed oils) can direct limited regulatory capacity toward the largest intersection of “risk × susceptibility,” thereby improving policy cost-effectiveness.

Overall, four advances in China’s domestic surveillance—greater granularity, broader matrix coverage, more quantitative exposure/risk characterization, and closer linkage with public-health practice—are shifting AFB_1_ governance from “post hoc inspection” toward a proactive cycle of “prospective hotspot identification → risk-tiered sampling → population linkage → policy evaluation.” When these surveillance gains are considered alongside the long-term Qidong evidence in which sustained biomarker declines coincided with reductions in HCC burden, a central conclusion emerges: in a country as climatically heterogeneous and dietarily diverse as China, localized and zoned surveillance is not an optional add-on, but a prerequisite for translating etiologic evidence into operational strategy. Accordingly, we recommend that China continue to strengthen a national capacity network for sample preparation and isotope-dilution LC–MS/MS, scale up the two-tier rapid screening–confirmatory system, promote P95/MOE-oriented risk communication, and adopt albumin-normalized AFB_1_–lys as a unified metric for cross-regional comparison, thereby providing a robust data foundation for the next cycle of standard revision and zoned governance [20,21,38].

## 6. Basic and Translational Research: From Mechanisms to Omics

China’s “basic-to-translational” trajectory in aflatoxin-related liver cancer (AFB_1_-HCC) can be summarized in three steps. First, at the levels of pathology and genomics, tumors arising from the joint influence of AFB_1_ exposure and HBV infection have been differentiated from “conventional” HCC. Second, multi-omics approaches have been used to systematically characterize pathway-level perturbations and chromosomal/lineage features that distinguish this etiologic subtype. Third, ongoing efforts aim to translate these molecular insights into traceable biomarkers and actionable intervention targets, as well as to link molecular readouts back to internal dose and clinical outcomes in human populations.

### 6.1. From Pathology and Mutational Signatures to Chromosomal Instability: Defining Disease Subtypes

Surgical specimens from high-incidence settings such as Guangxi, Shanghai, and Qidong supported early applications of array comparative genomic hybridization (aCGH) and differential proteomics. In the HBV(+)/AFB_1_(+) “dual-exposure” subtype, beyond the canonical TP53 R249S hotspot, tumors more frequently exhibited 8q gain and 17p loss, together with upregulation of proteins related to Wnt signaling, angiogenesis, and cell-cycle control, consistent with a lineage characterized by genomic instability coupled with convergent oncogenic signaling [56]. These molecular and chromosomal features align with the geographic enrichment of R249S observed in high-exposure regions [14,15,29].

Larger-scale population genomics further indicates that approximately ~10% of HCC in China can be recognized as an “AFB_1_-type” based on mutational signatures, and that this subtype is associated with elevated PD-L1 expression and increased microvessel density, suggesting that aflatoxin exposure may accelerate tumor progression through immune and vascular pathways [57].

### 6.2. RNA-Seq, Epigenetics, and Multi-Omics: From ‘Seeing Differences’ to Understanding Processes

Animal and cellular models have pushed the aflatoxin carcinogenesis timeline upstream into pre-neoplastic stages. In rats, RNA-seq data indicate that, beyond xenobiotic metabolism and redox pathways, alternative splicing, mRNA processing, and widespread dysregulation of lncRNAs are also perturbed, suggesting that AFB_1_-driven carcinogenesis is not limited to a single “DNA adduct → point mutation” route but involves broader systems-level disruption at the post-transcriptional regulatory layer [58]. In a dose–response model in ducklings, hierarchical changes in phase I/II metabolism, lipid metabolism, and cell-cycle/apoptosis programs were detectable before overt tumors formed, implying that transcriptomic signals may precede histopathologic alterations and thus hold potential for early warning [54].

Subsequent work has incorporated an inflammation–epigenetic axis. In the context of HBV, AFB_1_ exposure has been linked to aberrant promoter methylation of tumor-suppressor genes alongside upregulation of inflammatory mediators. Moreover, the aryl hydrocarbon receptor (AHR) has been identified as a key mediator of AFB_1_ toxicity and HCC development, with evidence of a pharmacologically tractable coupling to immune checkpoint pathways, supporting a molecular bridge between “chemical exposure” and “immune escape” that may be exploitable for prevention or interception strategies [59].

### 6.3. Practical Bottlenecks in Bridging Mechanism and Population

Despite a steadily strengthening evidence chain, full translation remains constrained by several real-world gaps. (1) Sample size and representativeness: truly informative “real-world” datasets—linking high-exposure populations to multi-year follow-up, obtaining HCC tissues, and integrating whole-genome/exome sequencing plus methylation and proteomics—remain scarce. (2) Insufficient longitudinal depth: within-person, time-resolved multi-omics evidence tracking the trajectory of high exposure → exposure reduction → (pre)neoplasia → tumor development is still limited, leaving a major gap in temporal inference. (3) Systems modeling that jointly accounts for co-exposures and HBV is still nascent: integrated modeling frameworks and coherent, sufficiently large domestic datasets are still developing, and the continuity of data streams needs strengthening [60,61]. (4) A limited toolbox for implementation: at the county level, deployable indicators remain largely restricted to AFB_1_–lys, urinary AFB_1_–N^7^–Gua, and circulating R249S [9,13,30,38]. Candidate signals emerging from multi-omics—such as lncRNA panels, splicing events, and methylation markers—have not yet completed the full translation loop of panel selection → multicenter validation → assay engineering for scalable use in community programs.

### 6.4. A ‘China-Adapted’ Translational Route

Building on China’s existing strengths in population cohorts and surveillance, we propose a China-adapted translational agenda with five priorities. (1) In regions such as Qidong, Guangxi, and western Guangdong, where historical biomarker data and retrievable tissues are available, implement spatial transcriptomics and single-cell profiling to test whether exposure-linked clones gain ecological dominance within the tumor microenvironment, as well as to benchmark these patterns against PD-L1 expression and microvessel density phenotypes [57]. (2) Link long-term AFB_1_–lys trajectories to mutational burden, copy-number variation, methylation subtypes, and immune infiltration to determine whether dose–epigenetic–immune thresholds or inflection points exist. (3) In the “post-HBV vaccination era,” establish prospective subcohorts of HBV-negative individuals who nevertheless experience fluctuating AFB_1_ exposure to identify potentially emerging metabolic–epigenetic subtypes and their risk trajectories. (4) Centered on AHR, DNA-repair nodes, R249S-related pathways, and candidate lncRNA/splicing events develop “small-but-high-yield” ddPCR/digital PCR and quantitative methylation panels, deployed in parallel with ID–LC–MS/MS, to create a three-dimensional surveillance framework integrating exposure–molecular–immune layers. (5) Incorporate co-exposures into an integrated evaluation that combines multi-omics with probabilistic exposure modeling, providing mixture-oriented evidence (“combined indicators”) to inform standard revision and inspection-site placement [24,38].

China has already largely achieved the transition from “pathology/genomics distinguishing AFB_1_-HCC” to “multi-omics elucidating its carcinogenic network.” A molecular portrait anchored by R249S, copy-number aberrations, and immune/vascular phenotypes, together with RNA-seq/epigenetic insights and the AHR axis, collectively outlines a chain from early exposure to tumor-ecosystem remodeling [54,56,57,58,59]. The next critical step is to align multi-omics signals with internal dose–biomarkers–outcomes, generating scalable molecular panels and evaluation tools that can be integrated with China’s existing infrastructures—circulating R249S testing, ID–LC–MS/MS capacity, and zoned surveillance networks—to complete a full translation loop from mechanistic understanding to precision prevention and control.

## 7. Regulatory Framework and Risk Management: From ‘Limits’ to ‘Governance’

Over the past two decades, China’s control of aflatoxins—particularly AFB_1_—has evolved from uniform maximum limits toward evidence-based, commodity-/scenario-specific regulation coupled with whole-chain governance. The current backbone standard, GB 2761-2017 [62](Maximum Levels of Mycotoxins in Foods), specifies AFB_1_ limits for peanuts and peanut products, maize and maize products, and selected vegetable oils (e.g., 20 μg/kg for peanut oil/maize oil; 10 μg/kg for several other vegetable oils), supported by the GB 5009 analytical method series as a unified basis for official inspections and enterprise self-testing [62,63]. This framework reflects a pragmatic baseline principle: stricter control for higher-risk commodities while maintaining feasibility.

### 7.1. Dynamic Optimization: “Evidence Feedback” and the 2025 Draft Revisions

Regulatory practice is increasingly shifting from “static limits” toward dynamic optimization informed by surveillance evidence. The 2025 draft revisions of GB 2761/GB 2762 [64] (released for public comment) propose refined limits for sensitive or newly emphasized contexts such as foods for special medical purposes (FSMP), nutritional supplements for older adults, and selected nuts and spices, signaling tighter alignment between national standards and domestic monitoring data. For international comparison, the European Union sets maximum levels for aflatoxins in groundnuts (peanuts) and processed products placed on the market for the final consumer at 2 μg/kg for AFB_1_ and 4 μg/kg for total aflatoxins, while groundnuts subject to sorting/physical treatment have higher limits (8/15 μg/kg for AFB_1_/total aflatoxins) (EU Regulation 2023/915) [65]. For maize, the EU specifies 5/10 μg/kg (AFB_1_/total aflatoxins) for maize (and rice) intended for sorting/physical treatment prior to retail, and 2/4 μg/kg for cereals and cereal-derived products [65]. In the United States, the FDA action level for total aflatoxins in foods, including peanuts/peanut products, is 20 ppb (μg/kg), providing an additional benchmark for risk management and enforcement [66].

### 7.2. International Alignment: Codex, the EU, and the U.S. FDA Action Level

Internationally, key references include the Codex General Standard for Contaminants and Toxins in Food and Feed (CXS 193-1995) and multiple Codes of Practice for prevention and reduction (e.g., peanuts CXC 55-2004, cereals CXC 51-2003, spices CXC 78-2017) [67,68,69,70]. These documents emphasize whole-chain control across production, storage, processing, and distribution through GAP/GMP/GSP/GDP plus HACCP, and they distinguish AFB_1_ from total aflatoxins, providing operational templates for national limits and governance procedures [65,66,67,68,69,70]. In the EU, Regulation (EU) 2023/915 replaced 1881/2006 from 2023 onward and applies commodity- and use-specific maximum levels; for groundnuts (peanuts) and processed products intended for direct human consumption, the limits are 2 μg/kg for AFB_1_ and 4 μg/kg for total aflatoxins, whereas for maize (and rice) intended for sorting/physical treatment prior to retail, the limits are 5/10 μg/kg (AFB_1_/total aflatoxins) (with higher limits applied to certain lots intended for further processing); the regulation also clarifies conversion principles for drying–rehydration and blending scenarios, informing China’s import/export compliance alignment and risk communication [65]. In the United States, the FDA commonly applies a 20 μg/kg action level for total aflatoxins in foods—similar in magnitude to some Chinese oil limits, though applied to different matrices [66]. We further note that regulatory frameworks also cover other staple commodities such as maize (corn) and rice, with limits defined by intended use (e.g., sorting/physical treatment vs. direct consumption) [66]. Edible oils (including corn oil) are typically managed under separate commodity categories because processing can alter toxin distribution and complicate representativeness, so surveillance and enforcement often rely on matrix-specific sampling and testing strategies [65,66].

### 7.3. Domestic Evidence Feeding Back into Standards: Risk Assessment and Regional Heterogeneity

China’s “risk assessment → standards feedback” pathway is increasingly supported by provincial and municipal monitoring. In Guangzhou and South China, MOE- and quantitative cancer risk-based assessments of market foods suggest overall risk is generally acceptable, yet high-exposure tail subpopulations require source control and process governance; these results provide quantitative support for refining limits and inspection frequency [42]. Complementary investigations of small workshops producing bulk/traditionally pressed peanut oil report high positivity rates and clear health-relevant signals, supporting regulatory prioritization of raw-material sorting, improved drying/storage, and workshop access/entry requirements [43]. Studies from western and inland regions further underscore regional heterogeneity—for example, Sichuan, China monitoring identified intermittently elevated AFB_1_ in dried chili and chili powder. Exceedance rates can be higher under EU thresholds than under Chinese thresholds, highlighting the potential need for commodity-specific limits or origin-based entry controls for spices and related products [71].

### 7.4. Whole-Chain Governance in Practice: From Upstream Control to Disposal and Traceability

“Paper limits” alone do not automatically translate into low population exposure; what matters is whole-chain governance. Upstream measures include rapid sorting and pre-storage screening for moisture, visible mold, and aflatoxins, together with systematic recording of storage temperature and humidity. At the processing stage, the most industry-ready options include (i) adsorptive decontamination using food-grade clay/mineral sorbents (e.g., bleaching earth and modified montmorillonite), which can be integrated into existing edible-oil refining lines; reports indicate that modified montmorillonite-based materials can reduce AFB_1_ levels in peanut oil, and (ii) alkali refining/neutralization and related refining steps, which may reduce aflatoxin carryover in certain oil matrices. In contrast, photocatalysis and other emerging technologies are currently more suitable for pilot-scale evaluation, given the requirements for specialized equipment, process validation, and regulatory acceptance. Together, these measures provide verifiable processing-stage options for managing over-limit raw oils. For enterprise self-testing and third-party capacity, higher-frequency screening and confirmatory testing are particularly important for foods intended for sensitive groups (e.g., FSMP, nutritional supplements for older adults). For disposal and traceability, rapid screening, electronic batch records, online delisting, and centralized destruction can be linked to contain long-tail risks [72].

### 7.5. Governance Beyond Enforcement: Quantifying “Institution → Exposure → Health” Links

The social-governance dimension of the control chain is also strengthening. In Guangzhou “high peanut oil consumption” communities, after implementation of local regulations for small workshops (SFWMR), interrupted time-series and mediation analyses suggest that strengthened workshop management and process control reduced the occurrence of liver-function abnormalities, with lower AFB_1_ levels in HMPO acting as an intermediate pathway, supporting a quantifiable “institution → exposure → health” relationship [73].

### 7.6. Toward Measurable, Controllable, and Evaluable Risk Reduction: Strategic Priorities

Overall, continued exposure reduction requires more refined standards and more verifiable process controls to sustain governance that is measurable, controllable, and evaluable, thereby compounding benefits with HBV vaccination and antiviral therapy and further lowering the AFB_1_-attributable fraction of liver cancer burden [65]. Priorities include the following:

(1) Advancing dynamic limits and differentiated sampling/inspection (e.g., temporary stricter thresholds or intensified inspection frequency in high-mold years or high-risk provinces);
(2) Integrating GB 2761 implementation in high-HBV-prevalence areas with secondary liver cancer prevention and nutritional substitution (“etiologic evidence → regulatory priority”);
(3) Aligning with international rules by issuing dual-track guidance for export-oriented peanuts/spices (domestic limits plus EU limits) to support compliance and risk communication;
(4) Institutionalizing an annual closed loop linking domestic surveillance, probabilistic risk assessment, and policy feedback [74].

## 8. Integrated Prevention Strategy: From Source Control to Individual Protection

Aflatoxin control should be organized around principles of being evidence-based, measurable, and routinely enforceable, and be translated into an integrated program spanning farm households, small workshops, high-risk individuals, and local governments. Such a program should be capable of stable operation in demonstration settings (e.g., Qidong) while remaining adaptable for scale-up across diverse contexts, including the mountainous Southwest, the South China coast, and the Huang–Huai Plain.

### 8.1. Source and Process Control: Stopping “Mold” in the Field and in Storage

The most cost-effective leverage point remains upstream and early post-harvest management. Priority actions include breeding (or introducing) peanut/maize cultivars with better storability (e.g., reduced shattering and improved kernel protection), rapid drying within ~24 h after harvest to safe moisture targets (peanuts in shell ≤ 9%; maize ≤ 13%), graded storage, and maintaining stability through low water activity + low temperature + ventilation. In coastal production areas and major storage hubs, incorporating cold-chain/low-temperature logistics into local grain-and-oil procurement and circulation rules can move mold control “upstream,” rather than relying only on endpoint maximum limits.

Field biological control using atoxigenic strains has been shown in multiple countries in Africa and the Americas to substantially reduce aflatoxin contamination in the field and/or during storage [75]. In China, standardized pilot programs could be implemented in major peanut-producing regions such as South China, Guangxi, and Hainan, applying locally adapted atoxigenic *A. flavus* strains as a source reduction strategy to relieve downstream processing and regulatory pressure [76,77,78]. In parallel, rapid pre-storage screening (moisture, visible mold, and aflatoxins) should be promoted, and online logging of warehouse temperature/humidity can be embedded into procurement contracts and insurance terms, creating an “quality control at intake” governance mechanism.

### 8.2. Process Detoxification Industry Governance and Standards Enforcement: Targeting the “High-Exposure Tail”

In China, residual AFB_1_ exposure is likely concentrated in the long tail of small workshops/small oil presses and self-produced/self-sold oils. Governance should therefore adopt a “two-wheel drive” strategy. First, technical enablement: standardize a simplified workflow—“drying → sorting → sealed storage → immunoassay-based rapid screening”—and provide low-cost consumables and practical training. Second, stricter sampling frequency: conduct 2–4 routine inspections per workshop per year, with an additional intensified round during hot–humid seasons; for positive lots, centralized refining or return of raw materials, forming a closed loop of “rapid screening → confirmatory testing → disposal/rectification”, are required [63,68]. Importantly, for aflatoxins, the dominant source of uncertainty is often sampling rather than analytical determination, because contamination is highly heterogeneous (“hotspots”); improper sampling can account for the majority of total error (reported to approach ~80% in some assessments). Therefore, sampling should follow established Codex/ISO principles (incremental sampling across lots, sufficiently large aggregate samples, comminution/homogenization, and controlled subsampling) [67,68,69,70].

In coordination with dynamic revisions of GB 2761, local regulations and filing standards could further require pre-market rapid testing for small workshops together with batch ledgers and traceability. A quantitative evaluation of regulation targeting traditionally pressed peanut oil from small workshops in Guangzhou reported significant improvements in product AFB_1_ levels and in liver-function abnormalities among high-consuming residents after implementation of the Small Workshop Management Regulation, supporting an “institution → exposure → health effect” pathway [79].

### 8.3. Population Health Promotion and Short-Course Interventions: Prioritizing Simple, Feasible Options

Community trials in Qidong and other high-exposure townships have shown that chlorophyllin and broccoli sprout beverages rich in sulforaphane [19], as well as dietary patterns emphasizing cruciferous vegetables plus dark leafy greens, can be implemented at the village level with low cost and good adherence, and they can be accompanied by measurable reductions in AFB_1_–DNA adducts [46,50,51]. On this basis, a three-tier rollout can be considered.

(1) Baseline promotion. In counties with high HBsAg prevalence, mold-prone staple risks, and a history of elevated liver cancer burden, implement practical dietary education: reduce intake of long-stored peanuts/maize flour; preferentially choose compliant packaged oils; and increase consumption of cruciferous and dark-green leafy vegetables.
(2) Seasonal/contextual intensification. During rainy seasons, post-typhoon periods, or historically high-contamination windows, provide 4–12-week short-course “detoxication support” for priority groups (preconception/pregnant women, children, patients with chronic liver disease, and HBsAg-positive individuals), using locally adapted options such as broccoli sprout powder formulations or chlorophyllin tablets.
(3) Clinical support. Incorporate a brief checklist of “recent suspected dietary exposure” into liver disease clinics and follow-up programs, enabling bidirectional feedback of exposure signals between clinical services and public health (CDC) systems, so that “controlling toxins” becomes an upstream component of disease prevention.

### 8.4. Precision Monitoring and Stratified Follow-Up: Deploying Costly Tests Where They Matter Most

A practical “three-tier biomarker toolbox” can be built around TP53 R249S (circulating DNA), serum AFB_1_–lys, and urinary AFB_1_–N^7^–Gua, with stratified use to maximize cost-effectiveness. At the primary-care level, conduct annual routine screening for HBsAg and ALT. For HBsAg-positive individuals with suspected dietary exposure, add AFB_1_–lys testing, using ELISA for initial screening where appropriate, with positive or high-value samples confirmed at provincial platforms by ID–LC–MS/MS. For those with persistently elevated AFB_1_–lys, proceed to R249S ddPCR testing. Individuals with the combined profile of HBsAg positivity + high AFB_1_–lys + R249S positivity can then be enrolled into a higher-intensity ultrasound/AFP surveillance pathway, thereby focusing secondary prevention on those at highest risk [58,80].

### 8.5. Public Policy and Health Equity: Ensuring Those Who “See Mold” Benefit First

Because AFB_1_ risk is jointly driven by climate and poverty, resource allocation should deliberately tilt toward vulnerable settings. We recommend that county-level budgets and agriculture-related programs prioritize practical infrastructure support—covered drying (drying areas), subsidies for plastic sheeting and sealed containers, village-level drying stations, and small low-temperature storage facilities. Extension services can disseminate a simple “six-step” decontamination workflow (select → shell/peel → wash → dry → grade → seal), and connect it to modern tools such as low-temperature storage, online monitoring of moisture/temperature, temperature-controlled logistics, and rapid immunochromatographic screening of raw materials [2,3,4,5].

In extreme-weather years or designated high-risk belts, implement differentiated inspection intensity and temporary stricter thresholds, and incorporate short-course interventions for high-exposure groups into public-health funding pools, so that “risk is dynamically escalated with climate.” This equity-oriented logic is consistent with the Qidong “natural experiment” trajectory (declining exposure followed by declining outcomes), and the resulting evidence can be fed back into policy design and prioritization [20,21,74].

### 8.6. Public Policy and Health Equity

Policy should prioritize interventions that disproportionately benefit households and communities most likely to be exposed—those facing high humidity and limited storage capacity, relying on informal supply chains (e.g., small oil presses), or living in HBV-endemic settings. Equity-sensitive allocation (infrastructure subsidies, technical extension, and risk-tailored inspection) improves both effectiveness and social legitimacy and helps ensure that exposure reduction is achieved where the preventable burden is greatest [20,21,79].

### 8.7. The ‘5+1’ Closed Loop and Implementation Guidance

We propose a practical “5+1” closed-loop framework for mold and aflatoxin control (Table 2), linking etiologic evidence to deployable governance actions and measurable endpoints. The five core components are as follows: (1) Source mold control (improved cultivars, rapid drying, low-temperature storage, and field biocontrol) [76,77,78]; (2) process detoxification (refining/adsorption, graded marketing, interception and re-processing of problematic lots); (3) tiered industry governance (routine and high-frequency “rapid screen → confirmation → disposal/rectification → trace-back” for small workshops) [62,63,67,78]; (4) short-course population “detoxication support” (chlorophyllin, broccoli sprout–based preparations, and feasible dietary patterns during seasonal peaks and in priority groups) [46,50,51]; (5) precision high-risk follow-up (HBsAg stratification, stepwise AFB_1_–lys testing, and R249S-triggered secondary prevention) [58,80]. We note that oltipraz is included here as a trial-based chemopreventive option evaluated with biomarker endpoints in selected high-exposure settings; accordingly, it has not been widely adopted as a routine population-level intervention.

The “+1” module is a climate-sensitive early-warning system, linking critical accumulated temperature (CAT) with crop production, storage, processing, and population risk signals as well as enabling differentiated inspection intensity and temporary thresholds in extreme years [81,82].

Together, this integrated strategy connects etiologic evidence (aflatoxin–liver cancer), methods and biomarkers (AFB_1_–lys/urinary adducts/R249S), the Qidong natural-experiment trajectory (declining exposure aligned with declining outcomes), and regulatory/governance instruments such as GB 2761 into a “measurable–controllable–evaluable” policy loop (Table 2). The central idea is to reduce systematic exposure upstream and midstream, lower individualized risk through targeted follow-up, and maintain resilience through climate-aware escalation, thereby further decreasing the fraction of liver cancer burden attributable to AFB_1_. These “5+1” implementation points can be incorporated into local technical guidance for mold control and aflatoxin risk management.

## 9. Implementation, Early Warning, Challenges, and Future Directions

### 9.1. Why ‘Look North and Upward’

Northeast and North China have traditionally relied on a relatively low-risk chain—cooler temperatures, rapid harvest, and timely drying—to limit aflatoxin formation. However, an increasingly common combination of drier growing seasons, high temperatures during crop maturation, and prolonged rainy spells during autumn harvest can expand the window for toxin-producing fungi in maize and peanuts, suggesting that previously “lower-risk” regions may no longer be intrinsically protected. In parallel, cross-province procurement of northern grains for southern feed supply chains can “spread out” localized upstream problems across downstream livestock belts in the Yangtze River Delta, Pearl River Delta, and Southwest China.

Recent multi-mycotoxin surveillance in China’s feed sector indicates substantial volatility at the raw-material stage and widespread co-contamination; although finished feeds are often kept within regulatory limits, upstream fluctuations argue that early warning and inspection need to move further upstream in the chain [53,83]. Global industry surveillance likewise reports that multi-mycotoxin co-occurrence is becoming the norm, with a persistently high proportion of 2024–2025 samples containing three or more mycotoxins, underscoring the need to consider potential additive/synergistic effects (e.g., with fumonisins) when evaluating the attributable carcinogenic burden of AFB_1_ [84,85].

### 9.2. Whole-Chain Integrated Surveillance and Early Warning: Cross-Matrix Transmission and Health Implications

In an era of high inter-annual volatility, monitoring should reconnect the evidence chain from upstream climate and storage signals to endpoint inspections, using risk-graded sampling and tiered analytics (rapid screening followed by confirmatory methods) to trigger timely action in grain/oil and feed supply chains [86,87]. Whole-chain models highlight that the “maize → dairy cattle → milk (AFM_1_)” pathway can transmit upstream AFB_1_ risk across regions via feed and cross-province distribution, supporting the need for integrated feed–food surveillance and early warning beyond traditional high-risk belts [86].

### 9.3. China’s Transferable Lessons and Global Contributions

The “long-term follow-up → biomarker evidence → policy intervention → outcome decline” chain established in Qidong and related settings offers a reusable template for adaptive governance under climate change scenarios. Structural measures such as staple/oil substitution (“switching grain/oil”), improvements in storage and processing with temperature-controlled mold prevention and detoxification, and biomarker-guided population actions centered on AFB_1_–lys (including short-course interventions where appropriate) can reduce internal dose within relatively short time windows and be accompanied by improvements in risk indicators [87].

By linking this paradigm to China’s large-scale feed–food multi-mycotoxin surveillance datasets, China can contribute “practice-based evidence” to international rule-making and technical guidance within FAO/INFOSAN/Codex frameworks, while also feeding insights back into the next cycle of national standard revision and capacity building [53,83,84,85,88].

### 9.4. A Realistic Policy Stance: Pessimism or Optimism?

Climate model projections alone point to a plausible “pessimistic” scenario in which inter-annual variability in AFB_1_ contamination increases. However, when combined with three practical levers—upstream substitution, whole-chain governance, and population biomarker stewardship—there is a credible pathway to push risk back into a controllable range. The core hazard is not an absence of technical options, but unseen volatility that escapes routine oversight.

**Figure 4:**
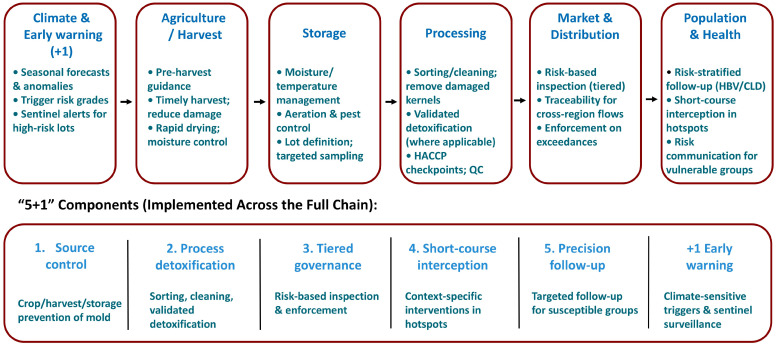
Operational logic of the climate-sensitive “5+1” framework across the climate–agriculture–storage–processing–population chain.

Accordingly, three actions should be prioritized: (1) operationalize an integrated early-warning network linking CAT → crops → storage → processing → population, enabling pre-positioned responses; (2) incorporate population biomarkers for AFB_1_ exposure into routine public-health monitoring and cross-validate them with food/feed inspection data; (3) implement differentiated inspection intensity and temporary, stricter provincial limits or sampling frequency schedules in extreme-weather years to match the realities of “high-variability years” [81,82,86,88,89,90,91]. Key recommendations are additionally summarized in Figure 4 and Table 3.

### 9.5. Persistent Bottlenecks and Future Directions

(1) China’s Major Evidence Chains and Global Relevance

China has established several internationally influential evidence chains linking aflatoxin exposure to primary hepatocellular carcinoma (HCC). First, exemplified by the Shanghai prospective nested case–control study and longitudinal follow-up in Qidong, China was among the early adopters of exposure biomarkers for population-level etiologic inference, demonstrating a clear association between internal dose and subsequent HCC risk. Second, in high-exposure settings, China pioneered prospective detection of the TP53 R249S “molecular fingerprint” in plasma/circulating DNA and aligned this signal with the characteristic mutational landscape of aflatoxin-related HCC. Third, community-based chemoprevention and dietary-interception trials using biomarker reduction as the principal endpoint (e.g., oltipraz, chlorophyllin, broccoli sprout beverages) produced directionally consistent intervention effects. Fourth, a policy-driven dietary transition—shifting staples from maize (and mold-prone oil crops) toward rice—paralleled long-term declines in population aflatoxin dose and HCC mortality, a rare example in the international etiologic literature of “policy → exposure → outcome” alignment [6,14,21,59,60,61].

On the basis of this evidence base, major international agencies such as IARC and EFSA have treated the joint effect of AFB_1_ exposure and chronic HBV infection on HCC as a highly credible chemical–viral dual risk factor, drawing substantially on population evidence from China—particularly from the Yangtze River Delta estuary and high-incidence belts in South and Southwest China—underscoring China’s central role in the global evidence system.

(2) Persistent Bottlenecks for Deeper Inference and Wider Implementation

Important bottlenecks remain for deeper inference and broader implementation, including (i) limited interoperability across meteorology–crop–storage–market–population data streams, (ii) uneven sampling representativeness under high spatial heterogeneity, and (iii) inconsistent cross-lab harmonization for key biomarkers and confirmatory analytics [89,92]. Addressing these gaps requires standardized protocols and shared data infrastructures [93,94]

(3) Priorities for the Next Phase: Systems, Standardization, and Zoned Prevention

Looking forward, priorities include building integrated early-warning systems (meteorology–crop–storage–market–population), strengthening standardization and inter-laboratory comparability, and implementing zoned prevention strategies that match region- and matrix-specific risk profiles [89,92,93,94].

## 10. Conclusions

This review synthesizes the full Chinese evidence chain linking aflatoxin (AFB_1_) to hepatocellular carcinoma (HCC) from discovery to prevention and control. Starting from early field investigations in high-incidence areas such as Qidong and Guangxi, China established urinary and blood-based exposure biomarkers and embedded them in prospective follow-ups, thereby quantifying the relationship between internal dose and subsequent disease risk. The etiologic inference was further strengthened by the TP53 R249S “molecular fingerprint,” which precisely connects an exogenous carcinogen to a characteristic tumor mutational pattern.

At the population level, China pioneered community-based chemoprevention and dietary-interception trials using biomarker reduction as the primary endpoint (e.g., oltipraz, chlorophyllin, broccoli sprout beverages). Together with a policy-driven dietary transition—from maize (and mold-prone oil crops) to rice—these efforts provide rare population-scale causal evidence consistent with a “declining exposure → declining outcome” trajectory. In recent years, province-level surveillance capacity based on immunoaffinity cleanup plus LC–MS/MS has clarified major grain/oil exposure pathways, regional and seasonal heterogeneity, and multi-mycotoxin co-exposure features, generating the data foundation for zoned governance and iterative standard refinement. Parallel advances in regulatory and methodological guidance (e.g., GB 2761 and EU performance requirements) are increasingly enabling governance that is measurable, comparable, and evaluable.

Looking ahead, key challenges include the following: completing an integrated “meteorology–crop–storage–market–population” early-warning system; accelerating inter-laboratory harmonization and mutual recognition for core biomarkers such as AFB_1_–lys; updating interaction and attribution models under changing HBV epidemiology and increasing mixture exposures; and institutionalizing process control and rapid testing capacity across the long tail of small workshops. We therefore recommend three parallel priorities: (1) technically, build a national climate-sensitive monitoring network with risk-based sampling; (2) methodologically, anchor harmonization on isotope-dilution LC–MS/MS with multicenter comparisons and unified reporting templates; (3) policy-wise, operationalize a zoned strategy—“source reduction → industry governance → short-course population interventions → stratified high-risk follow-up”—supported by digital enforcement, cold-chain logistics, and low-water-activity storage capacity.

In sum, China’s AFB_1_–HCC research has laid the evidentiary foundation of the “past” and is producing tangible prevention impact in the “present.” If upstream early warning, midstream governance, and downstream individual protection can be effectively integrated—while compounding the long-term benefits of HBV vaccination and antiviral therapy—China should be well positioned to further reduce the fraction of HCC burden attributable to AFB_1_.

## Figures and Tables

**Table 1:** Regional comparison of aflatoxin exposure evidence and liver cancer context across China.

Region (Province; Example Settings)	Dominant Exposure Matrices	Exposure/ Contamination Pattern (Qualitative)	Human Biomarker Evidence (Examples)	HCC burden/ Co-Factor Context (Qualitative)	Evidence Type Along Chain *	Evidence/Sources
**East China—Jiangsu** **(Qidong)**	Historically maize/corn products; later diversified diet; broader food-chain governance	Historically High, substantially declined after staple shift and mitigation	Urinary AFB_1_–N^7^–Gua; serum AF-albumin adducts (also used as intermediate endpoints)	Historically high HCC with HBV synergy; long-term prevention evidence	B+I+P	[6,19,50]
**South China—Guangxi (Fusui/Nanning)**	Corn porridge/maize; mixed staples	High in classic high-risk counties; intra-province heterogeneity	Serum AF-albumin adducts; urinary AFM_1_ and AFB_1_–N^7^–Gua in exposure studies	High HCC in high-risk counties; HBV prevalent	B+M	[11,51,52]
**East China—Shanghai** **(urban cohort)**	Mixed diet; background-level exposure	Low–Moderate (background)	Urinary AF biomarkers (including AF-DNA adduct marker) used in prospective risk study	HCC risk evaluated prospectively with biomarkers	B	[13]
**South China—Guangdong** **(peanut oil hotspot settings)**	Home-made/ unpackaged peanut oil, peanuts	Moderate–High (hotspots), especially in informal oil supply	Primarily exposure/risk studies; biomarker work reported in some settings (varies by study)	HBV synergy highlighted in local risk assessments	S+B	[42,43]
**Southwest—** **Sichuan (Chengdu**	Mixed staples; some dairy AFM_1_ signals reported	Generally Low– Moderate	Serum AF-albumin adducts (comparative population)	Moderate HCC context (lower than Fusui in comparative report)	B	[41,52]
**Central–North Huang–Huai Plain** **(Henan/Anhui)**	Maize/corn (also peanuts in some areas)	Episodic–Moderate; climate/storage-driven spikes	Large-scale food/feed monitoring; limited population biomarker cohorts published compared with south	Large population; HBV prevalence varies; “not inherently safe zone” framing supported by monitoring	S	[25,53]
**North China—Hebei/Shandong/Henan**	Maize kernels, feed/raw materials	Low–Moderate, but occasional high events (heterogeneous distribution)	Mainly feed/raw-material monitoring; human biomarker data less common in open literature	Food chain relevance (animal feed → AFM_1_ in milk)	S	[53,54]
**North/East—Shandong** **(peanut-** **producing)**	Peanuts, stored maize	Generally Low– Moderate for AFB_1_ in some surveys (context-specific)	Mostly dietary exposure/risk assessment (not biomarker-based)	Large production base; risk characterized in case studies	S	[55]
**Multi-zone dairy chain (cross-region; AFM_1_ as sentinel)**	Milk/dairy (AFM_1_), feed AFB_1_	Variable by climate zone/season	AFM_1_ surveys in milk; indicates upstream feed contamination	More about food-chain surveillance than direct HCC linkage	S	[56,57]

* Note: S = surveillance/monitoring (food/feed/milk); B = human biomarker evidence (e.g., AFB_1_–N^7^–Gua, AF-albumin adducts, AFB_1_-lys, AFM_1_); M = molecular pathology signatures (e.g., TP53 R249S); I = intervention trials with biomarkers as intermediate endpoints (e.g., chlorophyllin/oltipraz in Qidong); P = policy/natural experiment style population-level mitigation (e.g., staple shift, governance).

**Table 2:** Integrated Aflatoxin Control “5+1”—Implementation Matrix.

Pillar	Key Actions	Lead/Partners	Trigger/Timing	Indicators and Thresholds	Evidence/Sources
**① Source control**	Resistant cultivars; quick drying ≤ 24 h; safe moisture (peanut ≤ 9%, maize ≤ 13%); low-temp and ventilated storage; field biocontrol with atoxigenic A. flavus	Farmers/Co-ops; Agriculture Bureau; Extension stations	Harvest → pre-storage; before humid season	% lots meeting moisture spec; storage temp/RH logs; biocontrol coverage	[62,63,78]
**② Process detox**	Oil refining/adsorption/light/alkali detox; batch segregation; graded sales; reprocess out-of-spec lots	Oil mills; SMEs; Food industry	Continuous; pre-market	% batches > limit reprocessed; post-process AFB_1_ (μg/kg)	[62,63,72]
**③ Tiered governance**	Rapid screen → confirm (ID–LC–MS/MS) → dispose → trace; pre-sale rapid testing for small workshops; batch ledger; 2–4×/y routine + +1× hot–humid	Market Supervision; Local CDC labs; SMEs	Routine and seasonal surge	Non-compliance rate; sampling frequency; recall/closure time	[42,62,63,72]
**④ Short-course intervention**	Chlorophyllin/chlorophyll; oltipraz (trial-based; biomarker endpoint; limited routine implementation); broccoli sprout beverage; diet: crucifers + dark leafy greens; priority groups: pregnant, children, CLD, HBsAg(+)	CDC/Township clinics; Hospitals	4–12 weeks in high-risk season	Δ urinary AFB_1_–N^7^–Gua; Δ serum AFB_1_–lys	[19,32,46,50,52]
**⑤ Precision follow-up**	Primary-care HBsAg/ALT annual; AFB_1_–lys ELISA screen → ID–LC–MS/MS confirm; persistent high → TP53 R249S ddPCR; enroll into high-freq US/AFP	Hospitals; CDC labs	Quarterly/annual per protocol	% high-risk captured; adherence; median TAT	[58,80]
**+** **⑥ Climate-sensitive early warning**	CAT–crop–storage–processing–population linked alerts; differential sampling and temporary tighter limits in extreme-weather years; joint actions (Agri/Market-Sup/CDC/Meteo)	Meteorology + Agriculture + Market Supervision + CDC	Forecast-based; extreme-weather years	Alert accuracy; surge-sampling coverage; exceedance reduction	[80,81]

Note: “5+1” denotes five core pillars plus one additional module (“+1”), i.e., a climate-sensitive early-warning component that supports and connects the five pillars.

**Table 3:** Action-oriented roadmap for implementing the climate-sensitive “5+1” framework (what to do, who acts, and evidence anchors).

Operational Node	What to Do	Who Acts	Evidence Anchors (Examples)
**Upstream source control** **(climate–crop–storage)**	Climate-triggered risk grading; pre-harvest/harvest guidance; rapid drying; moisture control; lot definition for high-risk batches	Meteorological + agricultural agencies; cooperatives; grain handlers	Early-warning indicators; moisture/temperature thresholds; seasonal risk spikes [89,92]
**Process-level detoxification** **& sorting**	Sorting/cleaning; removal of damaged kernels; validated detoxification where appropriate; HACCP checkpoints	Processors; feed mills; edible-oil producers; QA/QC labs	Whole-chain monitoring and co-contamination signals [86]
**Tiered governance** **& compliance**	Risk-based inspection (focus on high-risk matrices/regions); enforcement on exceedances; traceability for cross-province distribution	Regulators; market supervision; third-party testing	Sentinel surveillance and targeted inspections improve efficiency [81,82]
**Short-course interception** **(high-risk people)**	Biomarker-informed targeting; short-course dietary interception/chemoprevention in high-exposure settings (context-specific)	Public health + clinical teams; community programs	Biomarker endpoints and field feasibility in sentinel sites [18,19]
**Precision follow-up** **for susceptible groups**	Risk-stratified follow-up for HBV carriers/CLD; combine exposure signals + clinical markers; intensified follow-up during high-risk seasons	Hospitals; CDC/public health; registries	Compound risk concept; interaction with host susceptibility [13]
**Cross-matrix synchronous assessment** **(multi-source exposure)**	Integrate grain/oil/feed/dairy data; harmonize sampling and analytics; rapid screening + LC–MS/MS confirmation; data platform for decision triggers	Inter-lab networks; surveillance programs; data platforms	Standardization and interoperable systems needed [93,94]

## Data Availability

No new data were created or analyzed in this study.
